# Exploring the Relationship Between Brain Neurochemistry, Cervical Impairments and Pain Sensitivity in People with Migraine, Whiplash-Headache, Low Back Pain and Healthy Controls: A Secondary Analysis of a Cross-Sectional Case-Control Study

**DOI:** 10.3390/jcm14051510

**Published:** 2025-02-24

**Authors:** Aimie L. Peek, Zhiqi Liang, Julia Treleaven, Trudy Rebbeck

**Affiliations:** 1 Sydney School of Health Sciences, Faculty of Medicine and Health, University of Sydney, Sydney, NSW 2006, Australia; trudy.rebbeck@sydney.edu.au; 2 School of Health and Rehabilitation Sciences, University of Queensland, St. Lucia, QLD 4072, Australia; l.zhiqi@uq.edu.au (Z.L.); julia.treleaven@uq.edu.au (J.T.); 3 John Walsh Centre for Rehabilitation Research, Kolling Institute, Northern Sydney Local Health District, St. Leonards, NSW 2064, Australia

**Keywords:** GABA, glutamate, neurochemistry, central sensitization, pain

## Abstract

**Background/Objectives**: Gamma-Aminobutyric Acid (GABA) and glutamate are the main inhibitory and excitatory neurochemicals of the central nervous system. Recently, increased GABA+ (GABA+ macromolecules) and Glx (glutamate and glutamine) levels have been reported in migraine. Conversely, decreased GABA+ and Glx levels have been reported in conditions such as chronic musculoskeletal pain and other chronic widespread pain conditions. This has led to the hypothesis that unique neurochemical profiles may underpin different headache and pain conditions. What is currently unknown is how neurochemical levels correlate with different clinical presentations of local and widespread pain sensitivity. The aims of this study were therefore to (i) explore the relationship between brain neurochemicals and clinical presentations of different headache and pain conditions and (ii) use a novel approach to explore how participants cluster based on their neurochemical profiles and explore the clinical characteristics of the participants in these neurochemical clusters. **Methods:** In this exploratory secondary analysis of a cross-sectional study, participants with migraine (*n* = 20), whiplash-headache (*n* = 20), and low back pain (*n* = 20), and healthy controls (*n* = 21) completed pain, disability and psychological distress questionnaires, received Magnetic Resonance Spectroscopy (MEGAPRESS), and underwent cervical musculoskeletal and quantitative sensory testing. Participants were classified based on cervical musculoskeletal impairment, increased cervical pain sensitivity, and central sensitization. Correlations between neurochemical levels and clinical classifications were explored. Cluster analysis was used to determine how participants grouped based on their neurochemical profiles. Pain, disability and psychological distress scores and clinical classifications were then compared between the resultant clusters. Post hoc testing explored increased cervical pain sensitivity within the clusters. **Results:** GABA+ levels moderately correlated with increased cervical pain sensitivity (r^2^ = 0.31, *p* = 0.006), with no other significant correlations. Cluster analysis revealed three neurochemical profiles, Cluster 1 (Low GABA+ levels) had moderate disability, Cluster 2 (highest Glx levels) had the lowest pain and disability, and Cluster 3 (highest GABA+ levels) had the highest pain and disability. Post hoc testing demonstrated that the cluster with the highest GABA+ levels (Cluster 3) had the most cervical pain sensitivity. **Conclusions:** This study suggests that considering the pain condition or presence of central sensitization alone is not sufficient to explain GABA+ and Glx levels. Our findings suggest that increased cervical pain sensitivity might be more reflective of GABA+ levels than pain condition or central sensitization and would benefit from further investigation to further elucidate the relationship between brain neurochemicals and clinical characteristics of pain sensitivity.

## 1. Introduction

A proposed mechanism of chronic pain involves the dysregulation between levels of Gamma-Aminobutyric Acid (GABA) and glutamate, the main respective inhibitory and excitatory neurochemicals of the central nervous system. It is believed that altered levels of GABA and glutamate are likely to result in central nervous system hyperexcitability, leading to central sensitization. However, the exact mechanism of this dysfunction is currently unknown. Several hypotheses have been generated, including a loss of GABAergic inhibition resulting in central sensitization [1], increased glutaminergic excitation leading to hyperexcitability [2,3], or, conversely, homeostatic increases in GABA as a result of or to counteract harmful increases in excitatory glutamate [4,5]. However, this relationship is complex given that excitatory glutamate is also a precursor to inhibitory GABA, thus making both inhibition and excitation interdependent [6].

There is increasing evidence, however, that the relationship between GABA and glutamate (the neurochemical profile) may differ between pain conditions. Our recent meta-analysis first suggested this by concluding that different pain conditions may have unique neurochemical profiles [7]. In particular, GABA+ (GABA+ macromolecules) and glutamate levels appear higher in studies of people with migraine compared to healthy controls [7,8,9,10], a neurochemical change which had not previously been observed in other musculoskeletal or pain conditions. Conversely, decreased GABA+ levels and increased Glx (a composite measure of glutamate and glutamine) are more commonly observed in chronic widespread pain conditions such as fibromyalgia [11] and neuropathic pain [1,12] compared with healthy controls. The limitations of these studies were that they did not directly compare different pain conditions. In an attempt to understand this, we conducted a direct comparison of brain neurochemical levels in different pain conditions, comprising migraine, whiplash-headache, low back pain, and healthy controls [13]. We found increased GABA+ in people with migraine and low back pain but not whiplash-headache when compared to their age- and sex-matched controls using a pair-wise analysis. These preliminary data suggest that unique neurochemical profiles may exist with different clinical pain presentations.

In order to better understand the relationship between brain neurochemical profiles and how these manifest in clinical presentations, this direct relationship needs to be studied. Previous studies have examined relationships between single neurochemicals and individual clinical tests of pain sensitivity. For example, moderate correlations have been demonstrated between pressure hyperalgesia and higher Glx levels [3] and lower GABA levels [11] in people with widespread pain conditions. Further, moderately positive correlations were found between GABA levels and Central Sensitisation Inventory scores (CSI) in people with migraine [14]. The limitations with these studies are not directly comparing pain conditions in the same study, only investigating GABA and glutamate (or Glx) individually, and only looking at individual tests of pain sensitivity rather than a whole clinical presentation.

A possible explanation for the difference in neurochemical profiles observed between migraine and other widespread chronic pain conditions could be the underlying pathophysiology. It has been proposed that increased GABA and increased glutamate observed in migraine are likely to increase the sensitivity of the trigeminal cervical nucleus (TCN) [10,15,16], a region known to play a role in relaying pain messages between the head and cervical spine in the pathophysiology of migraine and headache conditions [17,18]. As a result, increased GABA and glutamate levels may be associated with increased pain sensitivity in the head and cervical spine in headache conditions (increased cervical pain sensitivity). Conversely, in chronic pain conditions such as fibromyalgia or chronic musculoskeletal pain, the observed imbalance of low inhibitory GABA and high excitatory glutamate has been proposed to produce persistent neuronal excitability of pain processing pathways resulting in widespread central sensitization [19,20]. Taken together, we may hypothesize that neurochemical profiles of people with increased cervical sensitivity are likely to differ from those with widespread central sensitization; however, this has yet to be investigated.

To address the gaps in evidence, our study aims to explore the relationship between GABA+ and Glx levels with comprehensive clinical presentations of increased cervical pain sensitivity and central sensitization in varying headache and chronic pain conditions. The clinical presentations comprise patient-reported outcomes with clinical and quantitative measures of pain sensitivity and musculoskeletal function, in people with migraine, whiplash-headache, low back pain and controls. The secondary aim was to use a novel approach to explore how participants naturally cluster based on their neurochemical profile, and then explore the clinical characteristics of the participants within these clusters.

Knowledge of how specific neurochemical profiles manifest clinically would substantially advance the current understanding of brain neurochemistry, allowing the translation of neurochemical findings into clinical practice. This knowledge could allow for better identification and differentiation of clinical conditions and take steps toward the provision of neurochemical targeted treatments.

## 2. Materials and Methods

### 2.1. Study Design

This study was an exploratory secondary analysis of a cross-sectional study conducted at the University of Sydney and Western Sydney Local Health District. Ethical approval was granted from the Western Sydney Local Health District (WSLHD) study number HREC/17/WMEAD/429. Written consent from all participants was gained in line with the declaration of Helsinki.

### 2.2. Participants

#### Inclusion/Exclusion Criteria

Participants were people with pain conditions including migraine, whiplash-headache, low back pain and age-matched healthy controls. The pain conditions were chosen a priori to ensure we included people with different headache classifications and people without headache but with musculoskeletal pain. This should enable different combinations of musculoskeletal impairment, increased cervical pain sensitivity and central sensitization.

Participants with migraine were included if they satisfied the criteria for chronic migraine [21], namely had experienced migraine for over 3 months for more than 15 headache days per month. Participants with whiplash-headache were included if they satisfied the ICHD-3 criteria for “persistent headache attributed to whiplash”, meaning headache commenced after whiplash injury and has persisted for 3 months. Participants with low back pain were included if they satisfied the criteria outlined by the International Association for the Study of Pain (IASP) for chronic secondary musculoskeletal pain [22]. Criteria include pain arising anywhere between the last thoracic spinous process to the first sacral spinous process and had experienced symptoms for over 3 months. Further inclusion criteria for pain participants were moderate self-reported disability defined as Headache Impact Test (HIT-6 score) score > 50/78 for people with headache and Oswestry Disability Index (ODI) score > 20/100 for people with low back pain. Healthy controls were included if they had not experienced a headache or pain condition in the preceding three months and had never had a pain condition which had lasted over three months.

Participants were excluded from the study if they had any contraindications to MRI such as pregnancy or metal implants, or had conditions that compromise Magnetic Resonance Spectroscopy (MRS) such as metal braces or took medications known to effect GABA levels, e.g., gabapentin, topiramate. Participants were also excluded if they had co-existent pain conditions common to the other groups (e.g., whiplash-headache and low back pain) or had pain secondary to a more serious or red flag diagnosis such as fracture or tumour.

### 2.3. Recruitment

Participants with pain conditions were recruited from both primary and secondary healthcare settings. These included general practice, physiotherapy and neurology clinics. Participants either responded to a flyer in the waiting room, or treating clinicians gained the consent of participants to be contacted by the research team. Eligibility was established over the telephone, and potential participants were provided written information about the study prior to providing written consent. Participants then completed baseline questionnaires evaluating self-reported measures of pain, disability and psychological well-being. They then attended an appointment for clinical and MRS examinations which were conducted on the same day.

### 2.4. Baseline Questionnaires

Participants completed commonly recommended and validated questionnaires to assess self-reported pain intensity, sensitivity, disability and psychological distress. Pain intensity was assessed using the numeric rating scale (NRS) (score range 0–100, with higher scores reflecting higher levels of pain). Pain sensitivity was recorded using the Central Sensitisation Inventory (CSI) scores, where scores over 40 reflect central sensitization [23]. Self-reported disability was assessed using The Headache Impact Test (HIT-6 [24]), Neck disability index (NDI [25]), and Oswestry Disability Index (ODI [26]) to measure symptoms specific to each pain group, headache, neck pain, and low back pain, respectively. The HIT-6 score range is from 36 to 78 with higher scores demonstrating higher headache related disability. The NDI and the Oswestry scores range from 0 to 50, with higher scores demonstrating higher levels of neck and back pain related disability. All participants completed the World Health Organisation Disability assessment schedule (WHO-DAS 2.0 [27]) to measure general self-reported disability, which ranges from 0 to 100%, with higher scores demonstrating higher disability. Finally, the Depression Anxiety and Stress Scale (DASS-21) [28] was used to determine psychological status with higher scores indicating greater distress.

### 2.5. Clinical Examination

The clinical examination was performed by an expert physiotherapist with over 16 years’ experience in managing complex headache and musculoskeletal pain conditions. The examination consisted of two components. Firstly, clinical cervical musculoskeletal tests were used to identify cervical musculoskeletal impairments and increased cervical pain sensitivity. Secondly, quantitative sensory testing and clinical pain sensitivity measures were used to identify clinical evidence of widespread central sensitization.

### 2.6. Clinical Cervical Musculoskeletal Tests

Clinical cervical musculoskeletal tests included cervical range of motion, flexion rotation test [29], passive accessory intervertebral movements [30], the cranio-cervical flexion test (CCFT) [31], neck flexor and extensor isometric muscle strength [32,33] and endurance tests [34]. Performance in these tests in terms of range, strength and holding time informed the classification of cervical musculoskeletal impairment, and pain reported during each of the cervical musculoskeletal tests was used to classify increased cervical pain sensitivity (refer to Section 2.8 Clinical classification). All of these tests are validated tests and have been used in our previous research [35,36].

### 2.7. Clinical Tests of Widespread Pain Sensitivity

Static pain sensitivity was assessed using pressure pain thresholds (PPTs), cold pain threshold (CPT), and ice pain threshold (IPT) following established protocols [36,37,38] at three body sites, the cervical spine, lumbar spine and distally on the tibialis anterior. The side of the test corresponded to the most symptomatic side of the participants symptoms and was applied at the most uncomfortable level as identified through passive accessory intervertebral movements or manual examination of the lower limb for tibialis anterior.

Dynamic pain sensitivity was assessed using wind up ratio (WUR) and conditioned pain modulation (CPM) following the Quantitative Sensory Testing (QST) protocol by the German Research Network on Neuropathic pain [37]. The WUR was established using a 256 mN pin prick stimulator on the forearm of the most symptomatic or dominant side. The perceived pain rating from a single stimulus (0- no pain to 10-worst imaginable pain) was compared with that of a series of 10 repeated stimuli applied to the same location after a one-minute rest. The test was repeated three times. The WUR was calculated by taking the mean pain score from the three series of 10 repeated stimuli, divided by the mean pain score of the three single stimuli [39]. Conditioned pain modulation (CPM) was performed in sitting, and three PPT measurements were taken on the tibialis anterior of the symptomatic or dominant side with 30 s between each measurement. The contralateral hand was then placed in the conditioning stimulus, ice bath, and repeated PPTs were taken on the tibialis anterior. The CPM score was calculated by the difference between test stimulus (pre CPM), minus the test stimulus after the conditioning stimulus (post CPM) [40]. Dysfunctional CPM was defined as an increase in pressure pain threshold during the conditioning stimulus compared to control stimulus [40].

### 2.8. Clinical Classification

Participants were classified using the following criteria which were developed using the current evidence base, expert masterclass [41] and the clinical consensus of expert specialist musculoskeletal physiotherapists. An evidence informed pragmatic approach was chosen to mirror best available clinical practice. Participants could be classified into more than one classification. The three clinical classifications were (1) cervical musculoskeletal impairment, (2) increased cervical pain sensitivity and (3) clinical evidence of central sensitization.

#### 2.8.1. Cervical Musculoskeletal Impairment

We classified people with cervical musculoskeletal impairment if they had at least 2/3 of the following: (1) one positive articular sign (NRS > 4) pain on a segment ipsilateral to reported symptoms, positive flexion rotation test); (2) a reduction in composite range of motion, (under 295° based on minimal expected normal range of movement in adult populations [42]); (3) at least one positive muscular impairment test (reduced CCFT [31], reduced cervical isometric strength or endurance compared to normative values). These criteria were initially established by Jull et al. [43,44] and used in several of our previous clinical studies [35,36,45].

#### 2.8.2. Increased Cervical Pain Sensitivity

We classified people with cervical pain sensitivity if pain was reproduced on at least 3 of the following tests (1) range of motion testing, (2) the Flexion Rotation Test (FRT) [29], (3) passive accessory intervertebral movement (PAIVM) of a relevant segment ipsilateral to reported symptoms (4) during CCFT, isometric strength or endurance testing. These criterion were established and described by Liang et al. [46].

#### 2.8.3. Clinical Evidence of Central Sensitization (Central Sensitization)

We classified people with clinical evidence of central sensitization if they had 2 or more positive static pain sensitivity tests (Reduced distal PPT, Reduced CPT or IPT), at least one positive dynamic test (Increased WUR or dysfunctional CPM) [47] and a CSI score > 40 [23]. These criteria were informed by masterclasses in identification of central sensitization in clinical populations [36,41]

### 2.9. Neurochemical Measures

The primary neurochemicals of interest were the main inhibitory and excitatory neurochemicals of the central nervous system, namely GABA and glutamate. Currently the most reliable method to report GABA is as GABA plus macromolecules (GABA+) [48]. Glutamate was measured as a composite of glutamate and glutamine known as Glx [49]. We measured these neurochemicals using our establish protocol, described in detail here (Peek et al. [13]). In brief all participants were scanned on a Siemens 3T Magnetom Prisma MRI scanner with a 64-channel head coil. Voxels were positioned in the posterior cingulate cortex (PCC, voxel size 25 (AP) × 40 (RL) × 25 (CC) mm^3^). The PCC is a region that has demonstrated structural [50], functional [51] and neurochemical [13] changes in migraine and pain conditions, potentially due to its role within the default mode network. Further, its large homogenous nature demonstrates the ability to produce high quality spectra using MRS. Both GABA and Glx levels were acquired using the following MEGA-PRESS sequence, TR = 200 ms; TE = 68 ms; 192 averages (96 ON, 96 OFF); 2048 data points; spectral width 200 Hz; editing pulse frequencies set to 1.9 ppm and 7.5 ppm for editing of GABA; editing pulse bandwidth 70 Hz. Water-unsuppressed MEGA-PRESS data (with water suppression RF pulses deactivated) were also acquired to perform eddy-current correction and water-scaled quantification. Data were post-processed using the open-source MATLAB-based analysis toolbox; Gannet 3.1 [52], described in detail here (Peek et al. [13]) for data processing procedures. To quantify GABA and Glx levels we accounted for the tissue composition of the voxel, as well as different water content and relaxation times for grey matter, white matter and cerebrospinal fluid and therefore, reported alpha-corrected GABA concentration estimates relative to the internal tissue water signal [53].

### 2.10. Statistical Analysis

Descriptive statistics (mean ± standard deviation) were used to summarize data for each pain condition. Normality was assessed via the Kolmogorov–Smirnov test. Group differences were analyzed using ANOVA for normally distributed data and Kruskal–Wallis tests otherwise. Proportions were compared with Chi-square tests. Bonferroni correction was then applied to adjust for multiple comparisons.

Pearson’s correlation coefficients (r) assessed associations between brain neurochemical levels and clinical measures of musculoskeletal impairment, increased cervical pain sensitivity, and central sensitization, with Bonferroni correction for multiple comparisons.

Cluster analysis, performed in R (v4.0.1), explored participant grouping based on neurochemical levels. GABA, Glx, and the GABA:Glx ratio were normalized and scaled before Euclidean distances were calculated. Ward’s hierarchical agglomerative clustering was used, with a restriction of two to four clusters, assessed via dendrogram visualization. Descriptive statistics characterized clusters based on neurochemical levels, patient-reported outcomes, and clinical findings. Bootstrap 95% confidence intervals (percentile method) addressed skewed data, and Kruskal–Wallis tests compared cluster differences.

## 3. Results

### 3.1. Clinical Characteristics

A total of 81 participants were included in this study, comprising 20 with migraine, 20 with whiplash-headache and 20 with low back pain and 21 healthy controls. We excluded 5 participants from the cluster analysis (migraine *n* = 3; whiplash *n* = 1; control *n* = 1) as their neurochemical levels were outlying following the normalization process (>2.5 SD from the mean) or artefacts were observed on visual inspection of the spectra. Each case was investigated independently by two researchers and only excluded if there was clinical ground (e.g., participant taking a medication that can affect GABA levels), or poor quality data (e.g., movement artefact, poor signal to noise). This approach ensures reported neurochemical levels are representative of the group and likely to accurately reflect the neurochemical level rather than result from poor spectral quality. Therefore, a total of 76 participants, 56 with pain conditions (migraine *n* = 17; whiplash-headache *n* = 19, low back pain *n* = 20) and 20 healthy controls were included in the final cluster analysis.

The clinical characteristics of each pain group including the demographics, pain characteristics, psychological status, are presented in Table 1 with additional patient-reported outcomes and clinical data presented in Supplementary Table S1. Participants with migraine had a considerably longer mean (SD) duration of symptoms (19.7 years ± 11.3) compared with whiplash-headache (2.89 ± 2.24) and low back pain (7.31 ± 6.67). Pain at time of scan was similar across the pain groups, although the whiplash group had higher CSI, disability scores, and psychological distress compared to both low back pain and migraine groups.

While group mean GABA+ levels appeared higher in the pain conditions (Migraine 4.87 SD 0.62; whiplash-headache 4.74 SD 0.43; low back pain 4.84 SD 0.47) compared to the healthy controls (4.68 SD 0.43), this did not reach statistical significance. Further, there was no statistical difference in GABA+ or Glx levels between migraine, whiplash-headache and low back pain.

### 3.2. Results of the Correlational Analysis

GABA+ levels demonstrated a fair positive correlation with the clinical classification of increased cervical pain sensitivity (GABA, *r* 0.312, *p* value 0.01). Glx levels demonstrated a weak negative correlation with pain level (NRS) in the last week (−0.24, 0.02), CSI (−0.25, 0.02) and WHODAS (−0.23, 0.04), however these were not significant following Bonferroni correction for multiple comparisons. The GABA/Glx ratio demonstrated a weak positive correlation with pain level (NRS) in the last week (0.25, 0.02) but this was also not significant after Bonferroni correction. There were no other correlations found (Table 2, Supplementary Table S2).

### 3.3. Cluster Analysis

**Figure 1:**
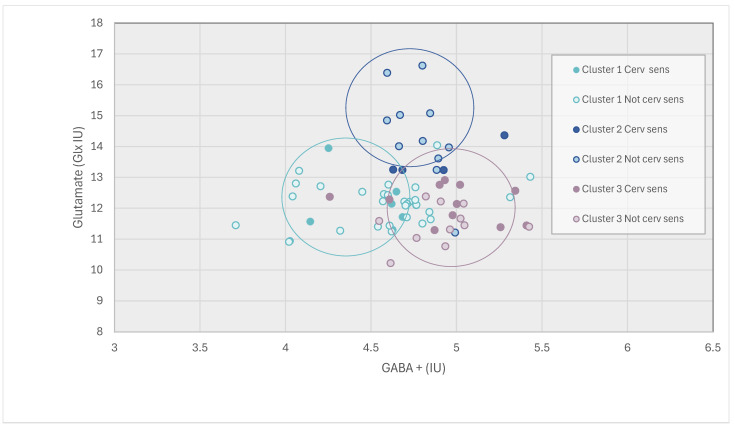
**Post Hoc Analysis:** Scatter graph of individuals GABA+ and Glx levels in relation to the presence of increased cervical pain sensitivity (Cerv Sens). Each individual participant is represented as a point on the plot in relation to their Glutamate level (IU) y axis, plotted against their GABA+ level x axis. Cluster 1 (Low GABA, Low Glx) is represented by green, Cluster 2 (High Glx, Low GABA) by blue, and Cluster 3 (High GABA, Low Glx) by purple. Those with increased cervical sensitivity are indicated with a dark fill, those without cervical sensitivity have a light fill. The overlayed circles demonstrate the position of each of the clusters on the graph. This graph shows the majority of those with increased cervical pain sensitivity have similar GABA+ and Glx levels to those in Cluster 3.

Three distinct groups were formed when participants were clustered by neurochemical levels (Agglomerative Coefficient = 0.96). The characteristics of the groups are described in Table 3. Participants with migraine, whiplash-headache, low back pain and healthy controls were distributed across all three neurochemical clusters (Table 2, Supplementary Table S3). Membership of each cluster are described below and illustrated in Figure 1.

**Neurochemical Cluster 1: Lowest GABA levels and low Glx levels.** This cluster had approximately equal proportion of people from each pain group and healthy controls. (23.7% migraine, 21.1% whiplash-headache, 26.3% low back pain, 28.9% healthy controls). Clinically these participants had moderate levels of pain, disability and psychological distress. This cluster had the highest proportion of people *without* any clinical classification (55.3%). [Post hoc proportion of cluster with increased cervical pain sensitivity 18.4% (lowest of all clusters); central sensitization 18.4%].

**Neurochemical Cluster 2: Highest Glx levels and low GABA levels.** This cluster had the highest percentage of healthy controls with twice as many healthy controls (40%) compared to any of the pain groups (migraine 20%, whiplash-headache 20%, low back pain 20%). Clinically these participants had the lowest pain levels, disability and psychological distress. Clinical classification suggested musculoskeletal impairment was the most prevalent classification within this group (40% compared with 26.7% increased cervical pain sensitivity, 13.3% central sensitization and 33.3% with no pain classification, Table 3 and Supplementary Table S3), i.e., during cervical musculoskeletal testing performance was limiting rather than pain limiting. [Post hoc proportion of cluster with increased cervical pain sensitivity 26.7%; central sensitization 13.3% (lowest of all clusters)].

**Neurochemical Cluster 3: Highest GABA+ level and low Glx.** This cluster had the lowest number of healthy controls (13%) and predominately consisted of musculoskeletal pain; whiplash-headache (34.8%) and low back pain (30.3%) with less people with migraine (21.7%). Clinically this cluster had the highest level of pain, disability and psychological distress compared to the other clusters. Clinical classification demonstrated that this cluster had the highest levels of musculoskeletal impairment, increased cervical pain sensitivity and central sensitization compared to the other clusters. Within this cluster there were more people with musculoskeletal impairment and increased cervical pain sensitivity than central sensitization. [Post hoc proportion of cluster with increased cervical pain sensitivity 52.2% (highest of all clusters); central sensitization 26.1% (highest of all clusters)].

### 3.4. Post Hoc Analysis

**Figure 2:**
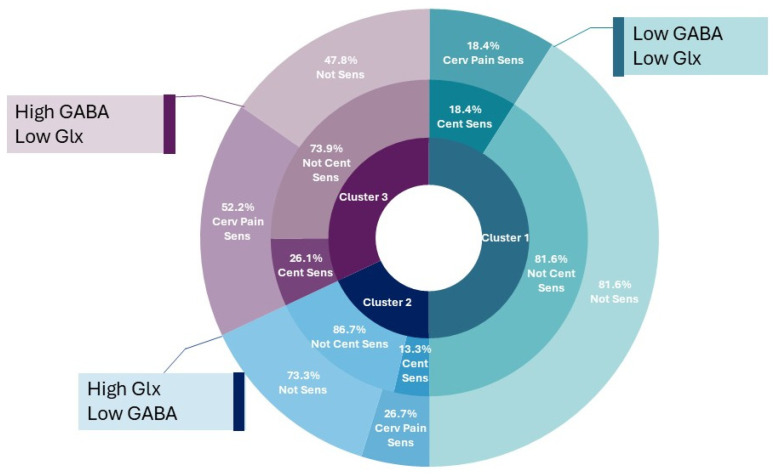
Infographic demonstrating prevalence of Increased cervical pain sensitivity (Cervical pain Sens) and central sensitization (Cent Sens) within the three clusters. Each cluster is represented by a colour (Cluster 1 Green, Cluster 2 Blue, Cluster 3 Purple. The inner ring shows the distribution of participants within each cluster. The middle ring shows the number of participants with central sensitization (dark colour) and the number without central sensitization (light colour). The outer ring shows the number of participants with increased cervical pain sensitivity (dark colour) and without cervical pain sensitivity (light colour). The boxes demonstrate the neurochemical profile of that cluster.

Increased cervical pain sensitivity was explored in a post hoc analysis following the observed correlation between GABA+ levels and increased cervical pain sensitivity in the whole group analysis. Results demonstrated higher levels of cervical pain sensitivity was present in Cluster 3 and the lowest level was present in Cluster 1 (Table 3). In the post hoc analysis the scatter plot in Figure 1 demonstrates the distribution of each individual participant with regard to their GABA+ and Glx level. Those with increased cervical pain sensitivity (circles with dark fill) sit closest to individuals in Cluster 3 regardless of their assigned cluster. Further, Figure 2 demonstrates the distribution of increased cervical pain sensitivity in relation to central sensitization in each of the clusters. In Cluster 1, 8 participants had central sensitization, and of those, just 2 also had increased cervical pain sensitivity. In Cluster 2, 6 participants had central sensitization, and of those, just 2 also had increased cervical pain sensitivity. However, in Cluster 3, the most pain-limited group, 6 participants had central sensitization, 5 of which also had increased cervical pain sensitivity. This finding demonstrates that the presence of increased cervical pain sensitivity typically occurred independently of the presence of central sensitization. One exception was in Cluster 3 (High GABA), where everyone who had increased cervical pain sensitivity also had central sensitization. It also provides further evidence to support the association between GABA+ levels and increased cervical pain sensitivity as illustrated in Cluster 3 High GABA Low Glx levels versus Cluster 1 Low GABA Low Glx levels (Table 3, Supplementary Table S3, Figure 1 and Figure 2).

## 4. Discussion

This study aimed to explore the relationship between brain neurochemical and clinical profiles. Several key findings emerged. Firstly, neurochemical levels appear to correlate with increased cervical pain sensitivity, but not central sensitization. Secondly, distinct clusters of neurochemical profiles existed in the chronic pain and control cohort, and when explored, each cluster had distinctly different clinical presentations.

This study found a fair positive correlation between GABA+ levels and increased cervical pain sensitivity, but not with central sensitization. Given the extensive body of basic and translational research suggesting that central sensitization arises from an imbalance between GABA and glutamate [1], we anticipated relationships between GABA+, Glx, and central sensitization. However, the only correlation observed was between GABA+ and increased cervical pain sensitivity. One possible explanation for this finding is that sensitization may be occurring at the level of the trigeminocervical nucleus (TCN), a region that converges input received from the trigeminal nerve and upper cervical spine nerves to produce an output within the brain [22]. This supports the theory that sensitization of the TCN through neurochemical imbalance may be a potential mechanism underlying migraine and headache disorders [54]. Further, targeting the desensitization of the TCN through the use of triptans has been shown effective in aborting migraines [55]. Therefore, a relationship between inhibitory GABA+ and increased cervical pain sensitivity is plausible and can be supported through some recent lines of enquiry.

Firstly, recent studies consistently report increased GABA+ levels in migraine and headache conditions, including pediatric and adult migraine, whiplash-associated headache, and whiplash with neuropathic pain [10,13,54,56]. This increase in GABA+ level has been attributed to sensitization of the trigeminocervical nucleus (TCN) and highlights the potential relevance of increased cervical pain sensitivity assessment in future neurochemical research. Secondly, in contrast, conditions involving widespread sensitization, such as fibromyalgia and spinal cord injury with neuropathic pain, show reduced GABA+ levels, suggesting distinct neurochemical mechanisms. Finally, while the relationship between clinical presentations of central sensitization and GABA+ levels remains relatively under-explored, evidence suggests that lower GABA+ levels may correlate with reduced pressure pain thresholds in fibromyalgia [11] and spinal cord injury with neuropathic pain [12]. Conversely, increased Central Sensitisation Inventory (CSI) scores correlate with elevated GABA+ levels in migraine, underscoring differing roles of GABA in localized versus widespread sensitization.

Results of our study also demonstrated that Glx levels had a weak negative correlation with pain and disability which were not observed with GABA+ (Supplementary Table S2). Although these results were no longer significant following correction for multiple comparisons, this same observation has been made in other studies of headache cohorts [57]. In contrast, a positive correlation between higher Glx levels and higher pain and disability has been documented in widespread pain populations, such as those with fibromyalgia [11,58,59]. These contrasting findings may reflect individual differences in neurochemical responses. In populations with low levels of central sensitization, as in this study (only >18.4% had clinical evidence of central sensitization), higher Glx levels may be counterbalanced by increased GABA+ levels, helping to prevent widespread pain sensitivity, as occurs in central sensitization. However, in more centrally sensitized populations, where higher Glx levels are associated with greater disability [11,58,59], Glx may not be matched by a corresponding increase in GABA+, leading to neural hyperexcitability and heightened pain sensitization. However, this relationship is likely to be complex, especially in whiplash, a condition with a wide variety of clinical manifestations including increased cervical pain sensitivity [60] in addition to widespread central sensitization. In summary, the direction of the relationship between Glx levels and disability appears to vary based on central sensitization levels, with GABA+ potentially moderating this relationship in less sensitized populations.

Three distinct clusters emerged from cluster analysis. Cluster 1 (low GABA+ levels) had moderate disability compared to the other clusters, Cluster 2 (highest Glx levels) had the lowest pain and disability, and Cluster 3 (highest GABA+ levels) had the highest pain and disability. Findings from Cluster 1 and 3 could be anticipated based on evidence in migraine, where positive correlations have been reported between increased GABA+ levels and increased CSI, longer migraine duration, and greater disability [10,14]. A finding not reported in other widespread pain conditions such as spinal cord injury, and chronic pain where lower GABA+ and higher Glx levels have been associated with greater pain and disability [11,12]. The difference between our study and other studies is not surprising given the high proportion of headache groups included within the study, and the low levels of central sensitization observed within the study population.

The low disability reported in Cluster 2 is more unexpected given that increased Glx is associated with greater disability and more pain sensitivity in both migraine and widespread conditions [3]. One explanation for this difference in neurochemical presentation could reflect variations in the brain regions and networks being studied. The posterior cingulate cortex, examined here, is a central node in the default mode network, which is most active during rest and helps restore homeostasis. In contrast, previous studies have focused on regions such as the insula, thalamus, and prefrontal cortex—areas associated with networks activated by pain stimuli and emotional pain processing. Thus, the roles of GABA and glutamate are also likely to differ depending on the brain region and network being studied and should be considered in making comparisons within the literature.

## 5. Strengths and Limitations

The strengths of this study are demonstrated through using new analysis techniques to interrogate the relationship between neurochemical levels and clinical characteristics in both headache and pain conditions. To our knowledge this is the first study to use pragmatic clinical assessment of pain sensitivity to bring better understanding to the meaning of brain neurochemical changes observed in the literature. This step is crucial in the early translation of evidence from basic science to clinical settings. Further, the novel use of the cluster analysis allows better insight into the neurochemical profiles of individuals rather than examining individual neurochemicals in isolation. However, some limitations exist. Firstly, this method of cluster analysis observes how individuals naturally cluster dependent on their neurochemical profile. Consequently, this led to groups being unevenly sized, ranging from *n* = 15, to *n* = 38, which can affect the statistical precision when comparing the groups, as analysis is primarily based on the smaller group size [61]. Secondly, the method used to perform clustering lead to the exclusion of a number of neurochemical outliers. A strength of this approach ensures that only high-quality data are included within the study; however, overzealous exclusions may lead to real data-driven findings being excluded from the study. Thirdly, Glx measurement is a composite of glutamate and glutamine, therefore the measure is not a direct measure of glutamate. Whilst this measurement is the accepted measure of glutamate within the field, most commonly used, and has been reliably quantified against known concentrations in vitro [62], there is acknowledgement as to how accurately it reflects glutamate levels [63]. Further, these data were extracted from MEGA-PRESS edited MRS spectrum, the gold standard measure for GABA, while it is commonly used to quantify Glx it is possible that PRESS data might be a better reflection of Glx [64]. Finally, the observational nature of this study elucidates potential new avenues for exploration; however, further research is required in larger independent cohorts to validate these neurochemical findings before they can be utilized to provide targeted treatment.

## 6. Conclusions and Clinical Implications

This study represents an important step toward translating neurochemical data into clinically relevant insights. The findings suggest that a pain condition or the presence of central sensitization alone is insufficient to fully explain variations in GABA+ and glutamate/Glx levels. Instead, the results highlight that increased cervical pain sensitivity may play a more critical role in influencing neurochemical levels, particularly in individuals with migraine, headache, or localized pain. In contrast, conditions characterized by widespread central sensitization, such as fibromyalgia, may exhibit distinct neurochemical responses. The clinical implication of such findings may in future expose different treatment options for these clinical manifestations of pain sensitivity. Specifically, clinical examination of pain sensitivity may identify individuals with particular neurochemical profiles who are likely to benefit from treatments that specifically target those aberrant neurochemical presentations. However, further validation is required. Overall, these findings underscore the need for future research to directly compare local trigeminocervical sensitization with widespread central sensitization to better understand the differential neurochemical mechanisms underlying these conditions.

## Figures and Tables

**Table 1:** Baseline characteristics of pain groups.

	Migraine (*n* = 20)	Whiplash-Headache (*n* = 20)	Low Back Pain (*n* = 20)	Healthy Controls (*n* = 21)	*p*-Value	Post Hoc Adj. Significance < 0.05
Age (years)	39.7 ± 10	42.3 ± 11.5	40 ± 13.8	38.2 ± 10.6		-
Sex (female *n*, %)	16, 80	14, 70	15, 75	16, 76.2		-
BMI	27 ± 6.5	28.4 ± 8.3	25.5 ± 6.2	25.1 ± 3.7		-
Educational level (University *n*, %)	12, 60	7, 41	8, 42.1	16, 72.7		-
**Pain Characteristics**						
Duration-years	19.7 ± 11.3	2.65 ± 2.1	7 ± 6.6	N/A	<0.001	W-M; LBP-M
Average pain intensity in last week (NRS 0–100)	66.1 ± 22.9	58.8 ± 21.4	55.6 ± 21.9	N/A	-	-
**Pain Sensitivity**						
Pain intensity at time of scan (NRS 0–100)	36.6 ± 30.2	40 ± 23.4	33.1 ± 22.7	4.3 ± 9.3	<0.001	LBP-C; W-C; M-C
CSI	48.6 ± 16.5	52.4 ± 18.7	31.3 ± 14.3	9.3 ± 9.6	<0.001	LBP-C; W-C; M-C; LBP-W
**Disability**						
WHODAS 2.0 (IQR)	26.9 ± 18.4	32.6 ± 19	20.5 ± 11.1	0.7 ± 1.6	<0.001	LBP-C; W-C; M-C
HIT-6	66.35 ± 6.6	63.71 ± 8.62	41.21 ± 5.42	32.27 ± 15.5	<0.001	W-C; M-C; W-LBP; LBP-M
**Psychological Status**						
DASS Total (IQR)	25.7 ± 20.6	42.3 ± 29.7	19.5 ± 15.7	4.1 ± 6.9	<0.001	LBP-C; W-C; M-C
**Neurochemical Level (IU)**						
GABA+ mean (SD)	4.87 (0.62)	4.74 (0.43)	4.84 (0.47)	4.68 (0.43)		-
Glx mean (SD)	12.79 (1.80)	12.00 (0.82)	12.23 (0.82)	12.81 (1.58)		-
GABA+/Glx mean (SD)	0.38 (0.05)	0.40 (0.04)	0.40 (0.04)	0.37 (0.05)		-
Clinical Classification *						
Cervical MSK impairment (*n*, %)	10 (50%)	18 (90%)	0 (0%)	0 (0%)		-
Increased cervical pain sensitivity (*n*, %)	8 (40%)	12 (60%)	4 (20%)	0 (0%)		-
Central Sensitization (*n*, %)	4 (20%)	9 (45%)	2 (11.1%)	0 (0%)		-

Mean and standard deviation are presented unless otherwise stated. * Participants could have more than one clinical classification and therefore percentage exceeds 100. Results following Bonferroni correction for multiple comparison are presented using the following acronyms: Whiplash-headache (W), Migraine (M), Low back pain (LBP), Control (C). N/A (not applicable) has been used where data for healthy controls were not collected.

**Table 2:** Correlations between neurochemical levels and clinical characteristics.

		Neurochemicals		
		GABA+ ^1^ (IU)	Glx (IU)	GABA/Glx (IU)
**C** **linical Classification**	Cervical MSK impairment	0.03	−0.09	0.10
	Central sensitization	0.10	−0.04	0.09
	Inc. Cervical pain sensitivity	0.31 **	−0.02	0.21
	MSK imp. and Inc. cervical pain sensitivity	0.18	−0.03	0.14
	All classifications	0.17	0.02	0.10
	None	−0.17	−0.07	−0.04

^1^ Neurochemical data are presented as an alpha corrected value, where GABA levels have been corrected depending on volume of grey and white matter within the voxel, with the assumption that GABA and Glx is present at the ratio of 2:1 in grey matter compared to white matter. ** indicates correlation is significant at the level of *p* < 0.004 following Bonferroni correction for multiple comparisons.

**Table 3:** Participant characteristics in Brain Neurochemical Clusters 1, 2 and 3.

	Cluster 1 (*n* = 38) Lowest GABA Low Glx	Cluster 2 (*n* = 15) Highest Glx Low GABA	Cluster 3 (*n* = 23) Highest GABA Low Glx	Post Hoc	
**Demographics**					
Age (years)	37.8 [34.0- 41.5]	39.4 [33.0–45.8]	43.0 [37.9–48.0]	-	
Sex (female *n*, %)	31 [81.6]	10 [66.7]	16 [69.6]	-	
BMI	26.0 [23.7–28.2]	26.2 [23.9–29.0]	27.7 [25.4–30.1]	-	
Duration (years)	7.6 [4.0–11.3]	6.2 [1.4–13.0]	7.7 [2.1–11.6]	-	
**Pain condition**					
Migraine (*n*, [%])	9/38 [23.7%]	3/15 [20%]	5/23 [21.7%]	-	
Whiplash-Headache (*n*, [%])	8/38 [21.1%]	3/15 [20%]	7/23 [30.4%]	-	
Low back pain (*n*, [%])	10/38 [26.3%]	3/15 [20%]	7/23 [30.4%]	-	
Healthy control (*n*, [%])	11/38 [28.9%]	6/15 [40%]	3/23 [12.1%]	-	
**Brain neurochemistry**					
GABA+ levels (IU)	4.5 [4.4–4.6]	4.8 [4.7–4.9]	5.1 [5.0–5.3]	1–2; 1–3; 2–3	
Glx levels (IU)	12 [11.8–12.3]	14.8 [13.8–14.9]	11.9 [11.5–12.1]	1–2; 2–3	
GABA/Glx ratio	0.38 [0.36–0.39]	0.34 [0.32–0.35]	0.43 [0.42–0.44]	1–2; 2–3; 1–3	
**Patient-reported outcome measures**					
Pain (NRS 0–100)	41.8 [30.8–53.5]	36.3 [17.4–55.3]	55.0 [40.6–68.2]	-	
DASS total (0–42)	15.0 [8.7–21.8]	11.3 [4.9–18.5]	19.3 [13.1–25.5]	-	
CSI (0–100)	36.7 [28.5–44.9]	26.5 [15.2–37.9]	37.6 [28.8–46.4]	-	
WHODAS (0–100)	19.8 [12.5–26.8]	15.9 [7.6–25.6]	23.2 [15.2–31.1]	-	
**Clinical classification ^**				**Total (*n* = 76)**	**Post hoc**
Cervical MSK Impair	9 [23.7%]	6 [40%]	11 [47.8%]	26 [34.2%]	-
Increased cervical pain sens.	7 [18.4%]	4 [26.7%]	12 [52.2%]	23 [30.2%]	-
Central Sensitization	7 [18.4%]	2 [13.3%]	6 [26.1%]	15 [19.7%]	-
No classification	21 [55.3%]	5 [33.3%]	8 [34.8%]	34 [44.7%]	-

All results presented as Mean [95% CI] unless otherwise stated, ^ Participants could occur in more than one of the groupings if satisfied multiple clinical classification criteria; therefore, percentages do not add up to 100 in rows or columns, there were no statistically significant differences in pain conditions, PROMS or clinical classifications between clusters.

## Data Availability

Raw data are available upon reasonable request.

## Supplementary Materials

The following supporting information can be downloaded at: https://www.mdpi.com/article/10.3390/jcm14051510/s1, Table S1: Clinical results across the pain groups. Demonstrating the clinical results used to inform the clinical classifications; Table S2: Correlation between neurochemicals and individual demographics and patient-reported outcomes; Table S3: Clinical results across the clusters. Demonstrating the clinical results and clinical classification within each of the clusters.

## Abbreviations

The following abbreviations are used in this manuscript, listed in order of appearance.

95% CI	95% Confidence Interval
ANOVA	Analysis of Variance
BMI	Body Mass Index
CCFT	Craniocervical Flexion Test
CFT	Cervical Flexion Test
CPM	Conditioned Pain Modulation
CPT	Cold Pain Threshold
CSI	Central Sensitization Inventory
DASS	Depression, Anxiety, and Stress Scales
FRT	Flexion Rotation Test
GABA	Gamma-Aminobutyric Acid
GABA+	Gamma-Aminobutyric Acid plus macromolecules
Glx	Composite of Glutamate and Glutamine
HIT-6	Headache Impact Test
IPT	Ice Pain Test
IQR	Interquartile Range
MEGA-PRESS	Mescher-Garwood Point-Resolved Spectroscopy
MRI	Magnetic Resonance Imaging
MRS	Magnetic Resonance Spectroscopy
MSK	Musculoskeletal
NDI	Neck Disability Index
NRS	Numeric Rating Scale
ODI	Oswestry Disability Index
PAIVM	Passive Accessory Intervertebral Movement
PCC	Posterior Cingulate Cortex
PPT	Pressure Pain Threshold
ROM	Range of movement
SD	Standard Deviation
WHODAS 2.0	World Health Organization Disability Assessment Schedule 2.0
WUR	Wind-Up Ratio
