# Supplementary material for: Exploring the Relationship Between Brain Neurochemistry, Cervical Impairments and Pain Sensitivity in People with Migraine, Whiplash-Headache, Low Back Pain and Healthy Controls: A Secondary Analysis of a Cross-Sectional Case-Control Study
Source: J Clin Med. 2025 Feb 24;14(5):1510. doi: 10.3390/jcm14051510 (PMC11899956; doi:10.3390/jcm14051510)
Supplement: Supplementary file 1 [file jcm-14-01510-s001.zip › jcm-3418848-supplementary.pdf]

Supplement S1

Table S1. Clinical results across the pain groups. Demonstrating the clinical results used to inform the clinical classifications.

	**Migraine (*n*=20)**	**Whiplash-headache (*n*=20)**	**Low back pain (*n*=20)**	**Healthy controls (*n*=21)**	**P value <0.05 after Bonferroni correction**
**Cervical musculoskeletal impairment**					
Pain on relevant cervical segment (*n*,%)	6 (30%)	14 (70%)	0 (0%)	0 (0%)	W-C; W-LBP; W-M
FRT +ve (*n*,%)	7 (35%)	16 (80%)	0 (0%)	0 (0%)	W-LBP; W-C; W-M
Composite ROM under 295 (*n*,%)	3 (15%)	11 (55%)	7 (35%)	0 (0%)	W-C; W-M
Reduced CCFT (*n*,%)	9 (45%)	14 (70%)	2 (10%)	2 (9.5%)	W-C; W-LBP;
Reduced isometric Flexion (*n*,%)	13 (68.4%*)	16 (80%)	8 (40%)	9 (45%*)	-
Reduced isometric Extension (*n*,%)	10 (52.6%*)	19 (90%)	10 (50%)	6 (30%*)	W-C;
Reduced Endurance cervical flexors (*n*, %)	10 (50%)	18 (90%)	5 (25%)	2 (9.5%)	W-C; W-LBP
Reduced Endurance cervical extensors (*n*, %)	13 (65%)	18 (90%)	12 (60%)	2 (9.5%)	LBP-C; M-C; W-C
**Cervical MSK impaired**	**10 (50%)**	**18 (90%)**	**0 (0%)**	**0 (0%)**	**LBP-M; M-C; W-LBP; W-C; W-M**
**Increased cervical pain sensitivity tests**					
Pain on ROM or FRT (*n*,%)	9 (45%)	13 (65%)	4 (20%)	0 (0%)	M-C; W-C; W-LBP

Pain on palp >4 segments >4/10 (*n*,%)	7 (35%)	11 (55%)	3 (15%)	3 (14.3%)	W-C
Pain limiting motor tests	13 (65%)	18 (90%)	12 (60%)	1 (4.7%)	W-C
**Increased Cervical Pain Sensitivity (*n*,%)**	**8 (40%)**	**12 (60%)**	**4 (20%)**	**0 (0%)**	**M-C; W-C**
**Central sensitisation tests**					
STATIC: Reduced PPT- distal (*n*,%)	12 (60%)	7 (35%)	14 (70%)	8 (38%)	-
Reduced CPT (*n*,%)	8 (40%)	9 (69.2%*)	11 (57.9%*)	10 (52.6%*)	-
Reduced IPT (*n*,%)	10 (50%)	8 (50%*)	10 (50%)	9 (40%*)	-
DYNAMIC: Reduced CPM (*n*,%)	3 (15%)	6 (37.5%*)	3 (15%)	4 (20%*)	-
WUR <1 (*n*,%)	7 (35%)	5 (31.3%*)	4 (20%)	6 (30%*)	-
CSI >40 (*n*,%)	11 (64.7%)	15 (46.9%)	5 (15.6%)	1 (3.1%)	M-C; W-C, LBP-C, W-LBP, M-LBP
**Central Sensitisation (*n*,%)**	**4 (20%)**	**9 (45%)**	**2 (11.1%)**	**0 (0%)**	**-**
**Brain neurochemicals**					
GABA+ level (IU)	4.87 (0.62)	4.74 (0.43)	4.84 (0.47)	4.68 (0.43)	-
Glx level (IU)	12.79 (1.80)	12.00 (0.82)	12.23 (0.82)	12.81 (1.58)	-
GABA+/Glx ratio (IU)	0.38 (0.05)	0.40 (0.04)	0.40 (0.04)	0.37 (0.05)	-
**Relevant disability**					
HIT-6	66.35 (6.60)	64.75 (8.72)	0.95 (5.40)	36.90 (10.04)	W-C; M-C; W-
NDI	15.00 (8.25)	22.90 (7.92)	7.80 (6.29)	1.05 (2.09)	LBP; M-LBP
Oswestry	12.19 (14.25)	26.49 (18.95)	22.30 (10.92)	1.66 (3.35)	M-C; W-C; W-LBP M-C;WAD-C; LBP-C;

***Adjusted percentage due to one missing value**

Supplement S2

Table S2. Correlations between neurochemical levels and demographics and pain measures.

		Neurochemicals		
		GABA+[1] (IU)	Glx (IU)	GABA+/Glx (IU)
**Demographics**	Age	0.12	-0.04	0.13
	Years with pain	0.05	0.03	0.01
**Pain measures**	Pain last week (NRS)	0.06	**-0.24 (0.04)**	**0.25 (0.02)**
	CSI	-0.10	**-0.25 (0.02)**	0.12
**Disability**	WHODAS	-0.06	**-0.23 (0.04)**	0.13
**Psychological**	DASS Total	-0.03	-0.18	0.11

[1] Neurochemical data are presented as an alpha corrected value, where GABA+ levels have been corrected depending on volume of grey and white matter within the voxel, with the assumption that GABA+ and Glx is present at the ratio of 2:1 in grey matter compared to white matter. Orange highlights demonstrating a weak correlation between Glx and pain measures (numeric rating scale [NRS] and Central Sensitization Inventory scale [CSI]) and WHODAS and GABA/Glx and NRS score, however, these were no longer significant following Bonferroni correction for multiple comparisons (p<0.004)

Table S3. Clinical results across the clusters. Demonstrating the clinical results and clinical classification within each of the clusters.

	**Cluster 1 (*n*=38)**	**Cluster 2 (*n*=15)**	**Cluster 3 (*n*=23)**	**P value <0.05 after Bonferroni correction**
**Cervical musculoskeletal impairment**				
Pain on relevant cervical segment (*n*[%])	10 [26.3%]	3 [20%]	10 [39.3%]	-
FRT +ve (*n*[%])	9 [25.6%]	4 [26.7%]	8 [34.8%]	-
Composite ROM under 295 (*n*[%])	6 [15.7%]	1 [6.6%]	12 [52.2%]	1-3; 2-3
Reduced CCFT (*n*[%])	12 [31.6%]	4 [26.7%]	9 [39%]	-
Reduced isometric Flexion (*n*[%])	21 [55.2%]	7 [46.7%]	15 [65.2%]	-
Reduced isometric Extension (*n*[%])	19 [50%]	6 [40%]	14 [60.9%]	-

Reduced Endurance cervical flexors (*n*[%])	18 [47.4%]	8 [53.3%]	15 [65.2%]	-
Reduced Endurance cervical extensors (*n*[%])	10 [26.3%]	3 [20%]	10 [39.3%]	-
**Cervical MSK impaired (*n* [%])**	**9 [23.7%]**	**6 [40%]**	**11 [47.8%]**	**-**
**Increased cervical pain sensitivity tests**				
Pain on ROM or FRT (*n*[%])	7 [18.4%]	4 [26.7.2%]	7 [30.4%]	-
Pain on palp >4 segments >4/10 (*n*[%])	8 [21.1%]	4 [26.7%]	11 [47.8%]	-
Pain limiting motor tests (*n*[%])	18 (47.4%)	7 (46.7%)	16 (69.6%)	-
**Increased Cervical Pain Sensitivity** (*n*[%])	**7 (18.4%)**	**4 (26.7%)**	**12 (52.2%)**	1-3
**Central sensitisation tests**				
STATIC:<br>Reduced PPT- distal (*n*[%])	22 [57.9%]	8 [53.3%]	11 [47.8%]	-
Reduced CPT (*n*[%])	22 [59.5%]	8 [57.1%]	8 [36.4%]	-
Reduced IPT (*n*[%])	15 [39.5%]	9 [60%]	12 [52.2%]	-
DYNAMIC:				
Reduced CPM (*n*[%])	7 [18.4%]	5 [33.3%]	4 [17.4%]	-
WUR <1 (*n*[%])	11 [28.9%]	6 [40%]	5 [21.7%]	-
CSI >40 (*n*[%])	16 [42%]	4 [33.3%]	12 [47.8%]	-
**Central Sensitisation (*n*[%])**	**7 [18.4%]**	**2 [13.3%]**	**6 [26.1%]**	**-**

* Participants can have more than one clinical classification within in this table
